# BandFocusNet: A Lightweight Model for Motor Imagery Classification of a Supernumerary Thumb in Virtual Reality

**DOI:** 10.1109/OJEMB.2025.3537760

**Published:** 2025-02-03

**Authors:** Haneen Alsuradi, Joseph Hong, Alireza Sarmadi, Robert Volcic, Hanan Salam, S. Farokh Atashzar, Farshad Khorrami, Mohamad Eid

**Affiliations:** Engineering DivisionNew York University Abu Dhabi 167632 Abu Dhabi 129188 UAE; Department of Electrical and Computer EngineeringNew York University 5894 New York NY 10012 USA; Division of ScienceNew York University Abu Dhabi 167632 Abu Dhabi 129188 UAE

**Keywords:** Brain-computer interface, deep learning, EEG, human augmentation, motor imagery, supernumerary robotic limbs

## Abstract

*Objective:* Human movement augmentation through supernumerary effectors is an emerging field of research. However, controlling these effectors remains challenging due to issues with agency, control, and synchronizing movements with natural limbs. A promising control strategy for supernumerary effectors involves utilizing electroencephalography (EEG) through motor imagery (MI) functions. In this work, we investigate whether MI activity associated with a supernumerary effector could be reliably differentiated from that of a natural one, thus addressing the concern of concurrency. Twenty subjects were recruited to participate in a two-fold experiment in which they observed movements of natural and supernumerary thumbs, then engaged in MI of the observed movements, conducted in a virtual reality setting. *Results:* A lightweight deep-learning model that accounts for the temporal, spatial and spectral nature of the EEG data is proposed and called BandFocusNet, achieving an average classification accuracy of 70.9% using the leave-one-subject-out cross validation method. The trustworthiness of the model is examined through explainability analysis, and influential regions-of-interests are cross-validated through event-related-spectral-perturbation (ERSPs) analysis. Explainability results showed the importance of the right and left frontal cortical regions, and ERSPs analysis showed an increase in the delta and theta powers in these regions during the MI of the natural thumb but not during the MI of the supernumerary thumb. *Conclusion:* Evidence in the literature indicates that such activation is observed during the MI of natural effectors, and its absence could be interpreted as a lack of embodiment of the supernumerary thumb.

## I. Introduction

Human movement augmentation, subject to centuries of human fascination and portrayal in science fiction genres, is now an emerging field of research in robotics and artificial intelligence (AI). It enables one to control supernumerary effectors, such as robotic limbs or fingers [1], which can move concurrently with the user's natural limbs. By increasing the number of movement degrees-of-freedom (DoFs) and extending capabilities beyond natural limits, human movement augmentation can bring enhanced dexterity and manipulation abilities, with a variety of applications in healthcare, industry, and specialized environments [2].

With the recent advances in human movement augmentation, electroencephalography (EEG) has also gained great interest as a potential mode of control in brain-computer interface (BCI) systems [3]. EEG, as a non-invasive technique, offers high temporal resolution, providing both convenience and online control with low latency, two important factors in the control of augmented devices [4]. In the context of a BCI system, EEG is often utilized to capture neural activity associated with motor execution (ME) and motor imagery (MI), observable through event related desynchronization (ERD) in the alpha band (9–13 Hz) at the motor cortex [5]. MI, in particular, has been utilized to control supernumerary effectors [6], [7].

EEG's potential is limited by its low spatial resolution [8] and susceptibility to noise, which affects the reliability and concurrency of control in EEG-based BCI systems [9]. This often results in EEG-based augmentations being controlled using the MI of actions already present in the natural body's repertoire, particularly those that involve larger body parts or movements, such as walking in a specific direction or grasping an object—- the neural activation associated with the MI of these actions are more easily detectable, thus improving reliability of control albeit at the expense of concurrency [10], [11], [12].

Recent developments in EEG signal decoding and classification [13], [14], [15], [16], as well as literature that support the ability to represent additional natural effectors through brain plasticity [1], [17], have inspired the mapping of a supernumerary effector's control to a distinct neural signature associated with the supernumerary effector itself. For example, Penaloza et al. demonstrated that participants could control an augmented third arm using neural activity based on MI of the supernumerary effector, even while their natural arms were engaged in a ball-balancing task [6], [18]. Their proposed BCI system used an automatic selection algorithm that measured the power spectral density (PSD) of EEG data from subjects, and triggered movements of the supernumerary effector when specific thresholds were crossed [6]. Additionally, a 4-week longitudinal study by Liu et al. provided further evidence towards the development of a distinct MI function that could be mapped to the control of a supernumerary thumb. A genetic algorithm was used to select optimal channels for each participant, achieving a within-subject classification accuracy of 70% in distinguishing between MI and rest periods using common spatial pattern (CSP) filters and a support vector machine (SVM) [7]; CSP based methods are common in traditional MI decoding [19], [20], [21]. A further study by Liu et al. demonstrated that using the same method, along with the same model and EEGNet [14], successfully classified MI signatures of a supernumerary finger, distinguishing them from MI signatures of right-hand movement and rest. The classification achieved accuracies of 86.3% and 88.2%, respectively, using k-fold cross-validation [22].

These studies show the potential of MI classification for EEG-based BCI control of supernumerary augmentations, but also bring new areas of consideration. For instance, the validity of the MI signature associated with the supernumerary effector should not be overlooked. Most literature compared the neural activity of the augmented effector to that of the resting state. While this is a valid way to distinguish the presence of MI, it is difficult to conclude that the MI activity is associated with the supernumerary effector without a comparison against the MI activity of a natural effector. Furthermore, the nature of this supernumerary effector should also be taken into account. Different natural effectors like fingers, arms, and legs elicit varying MI patterns [23]. Similarly, it is plausible to consider that the nature of the supernumerary effector, whether this be its appearance or function, will also affect the MI signature associated with its movement. For a supernumerary finger, the best point of comparison to show evidence of this would be the MI signature associated to the movement of a natural finger with similar appearance or function to the supernumerary one.

Another point of consideration is the model used to perform the classification of the EEG activity, as well as its evaluation. While there are various EEG classification models that provide high accuracy classifications, they are typically developed to be flexible across various EEG datasets [14], [15]. As a result, they often possess a large number of parameters, which in turn affect training and inference times, two important factors in the context of BCI. Furthermore, the ability for such a model to generalize across subjects is also important when considering a real-world application of EEG-based BCI. Given the personalized nature of neural activity across individuals [3], it is difficult to create a one-size-fits-all solution, but a model that can generalize well across subjects would allow the use of augmentations without extensive training. Similarly, when evaluating the accuracy of the model, while methods such as *k*-fold cross-validation provide a reliable estimate of model performance across a combined dataset, when considering the application of the model in BCI, where individual variability can significantly affect model performance, a leave-one-subject-out cross-validation method is a more realistic measure of how well a model can generalize to new subjects. Despite this being a standard evaluation technique for the generalizability of ML models, most of the work done in this domain utilizes either within subject classification [7], [18] or k-fold cross-validation [22].

Building upon this understanding of EEG-based BCI systems for controlling supernumerary effectors, we aim to investigate whether MI activity associated with a supernumerary effector can be reliably differentiated from that of a natural one of similar appearance and function. Furthermore, given the limitations of existing classification solutions, we explore alternative approaches that ensure not only accuracy, but also speed and trustworthiness.

Thus, in this study, we use an EEG dataset from an experiment where we designed a virtual reality (VR)-based paradigm in which twenty subjects observed the flexion or extension of either their virtual natural thumb or a supernumerary thumb (attached to their virtual right hand), followed by a request to perform MI of the observed movement during which their EEG data were collected [9]. We propose a lightweight deep learning model that incorporates temporal, spectral, and spatial features of EEG data to classify the MI of the natural vs. supernumerary thumb. Our model extracts features from three spectral bands, namely, the delta, theta and alpha bands, and aggregates the extracted features across the channel dimension. The model employs a channel attention mechanism to dynamically highlight the most relevant bands towards the classification task. Our model outperforms more complex state-of-the-art EEG-based deep learning models. We employ leave-one-subject-out cross-validation, allowing us to assess the model's performance on previously unseen subjects. We further validate the model's reliability using explainability analysis through Shapley values [24], identifying the most influential features used for predictions [25]. These features (i.e., cortical regions) were cross-validated with event-related spectral perturbation (ERSP) analysis and existing literature, confirming the involvement of these regions in MI and supporting the model's trustworthiness.

## II. Materials and Methods

Please refer to the Supplementary Materials for the details on material and methods, including the experimental setup, proposed model architecture, data processing and explianability analysis.

## III. Results

### A. Classification

**Fig. 1.**
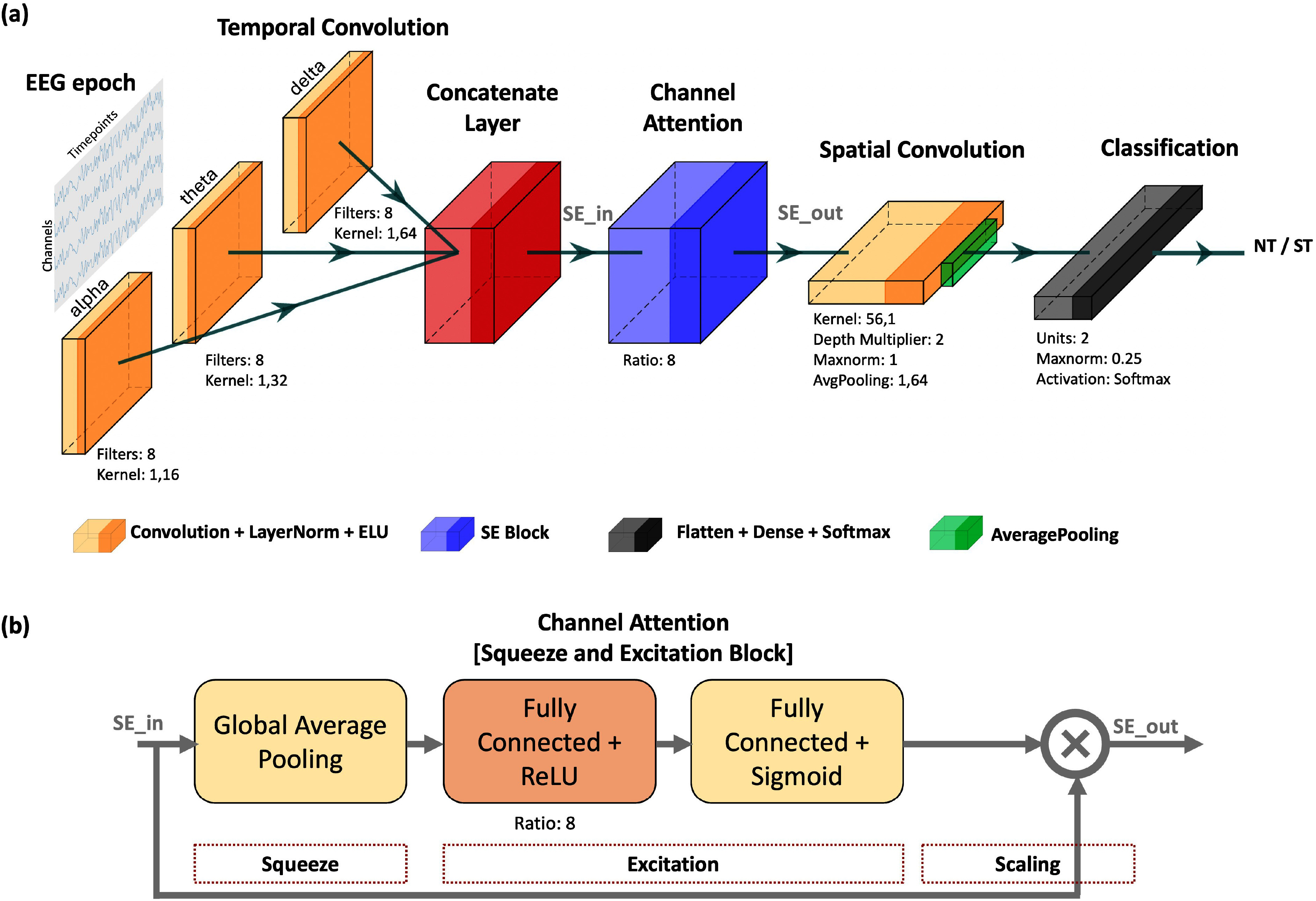
BandFocusNet model. (a) The architecture of the proposed model, with a breakdown of the role of each block. (b) An illustration of the channel attention block (squeeze-and-excitation).

Baseline models, as well as BandFocusNet (see Fig. 1, were trained and validated using the leave-one-subject-out cross-validation technique. Table I lists the performance of each model on the test subject and highlights the highest accuracy for each subject. EEGConformer achieves an average accuracy of 67.42%, CapsuleNet achieves an average accuracy of 67.51%, ATCNet achieves an average accuracy of 68.90%, and BandFocusNet achieves an average accuracy of 70.90%. Furthermore, the results indicate that BandFocusNet achieves the highest average accuracy while using the fewest parameters—at least 3.5 times fewer than the next most complex baseline model, ATCNet (see Table II).

**TABLE I:** The Performance of the Baseline Models and BandFocusNet Under Leave-One-Subject-Out Evaluation

**Subject/Model**	**EEGConformer [15]**	**CapsuleNet [13]**	**ATCNet [16]**	**BandFocusNet (Proposed)**
**1**	**90.08**	87.02	89.31	86.26
**2**	53.04	**71.30**	60.00	70.43
**3**	65.83	62.50	**70.00**	69.17
**4**	76.22	73.43	74.83	**78.32**
**6**	59.84	60.63	61.42	**66.14**
**7**	71.82	70.00	70.00	**75.45**
**8**	59.23	58.46	58.46	**64.62**
**9**	58.78	**59.54**	58.78	57.25
**10**	69.77	68.60	**73.26**	67.44
**12**	58.12	58.12	**60.68**	**60.68**
**13**	**59.41**	**59.41**	56.44	**59.41**
**14**	70.45	67.42	72.73	**76.52**
**15**	60.31	**69.47**	61.83	66.41
**16**	75.61	63.41	78.05	**80.49**
**17**	80.28	**84.51**	83.10	82.39
**18**	69.91	66.37	**73.45**	**73.45**
**Average**	67.42 $\pm$ 9.91	67.51 $\pm$ 8.64	68.90 $\pm$ 9.71	**70.90 $\pm$ 8.54**

The Accuracy for Each of the Subjects is Listed When It is Considered as the Test Set.

**TABLE II:** Comparison of the Number of Parameters Between the Baseline Models and BandFocusNet

**Model**	**#Parameters**
EEGConformer [15]	843,873
CapsuleNet [13]	746,880
ATCNet [16]	187,289
BandFocusNet (Proposed)	**47,908**

### B. Explainability

**Fig. 2.**
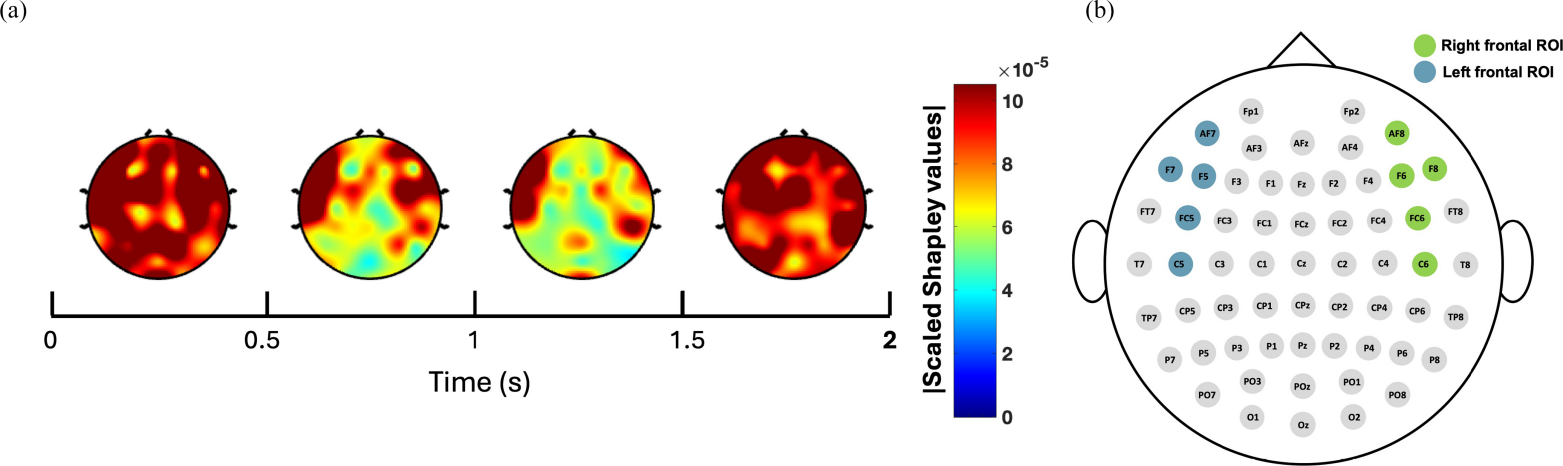
Explainability of BandFocusNet. (a) Time-course topography plots of the absolute scaled Shapley values during the MI period. (b) Defined ROIs based on the extracted Shapley values.

Fig. 2(a) presents a topographical representation of the absolute scaled Shapley values, with the highest values indicating the most influential channels. Overall, the model appears to utilize most EEG channels in its predictions. However, the frontal region, both left and right, demonstrates higher influence throughout the MI epoch. Therefore, as shown in Fig. 2(b), we define two regions of interest (ROIs)—the right and left frontal regions—for further analysis and validation. The right ROI consists of (AF8, F6, F8, FC6, and C6) whereas the left ROI consists of (AF7, F5, F7, FC5, and C5).

### C. Neural Cross-Validation

**Fig. 3.**
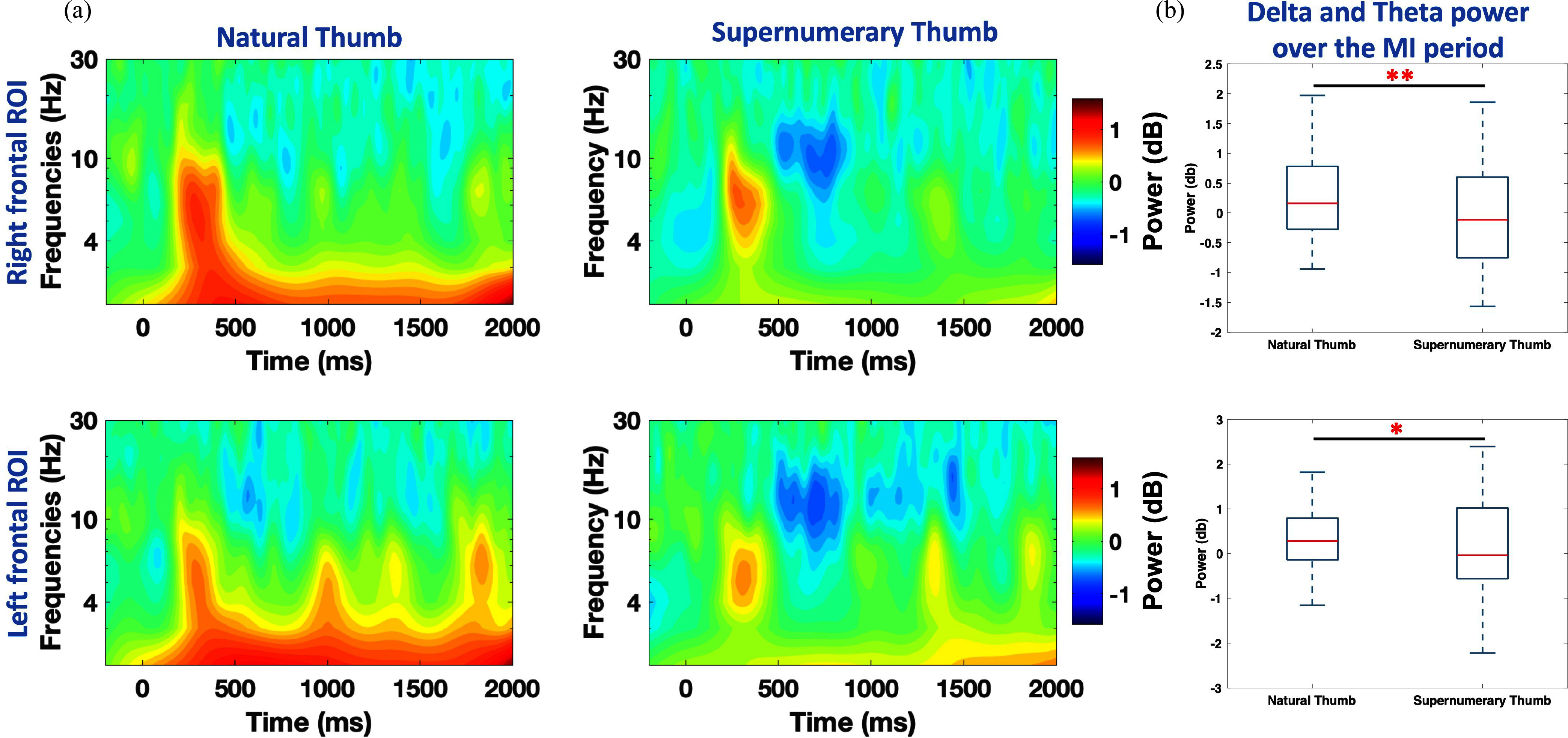
Neural cross-validation. (a) ERSPs for the right and left frontal ROIs during the MI of the NT and ST conditions. (b) Boxplot of the average power in the delta/theta bands during the MI period, comparing NT and ST across both ROIs.

We performed neural cross-validation and examined the ERSPs of the defined ROIs based on the Shapley values analysis. Fig. 3(a) shows the ERSP heatmaps for both ROIs under the natural thumb MI and the supernumerary thumb MI conditions. This examination revealed an event-related synchronization in the delta and theta bands in both ROIs during the natural thumb MI. This activation was not observed during the MI of the supernumerary thumb. Fig. 3(b) presents a boxplot comparing the average power (in dB) between the natural thumb MI and the supernumerary thumb MI in the delta and theta bands across both ROIs, highlighting a statistically significant difference in the delta/theta average power between the natural thumb MI and supernumerary thumb MI conditions for both ROIs (${\mathit{p}} = 0.013$ (Left frontal), ${\mathit{p}} = 0.001$ (Right frontal); bootstrap test; Bonferroni correction; N=16 samples). Thus, both ROIs exhibit statistically significant differences in their encoding of the imagined thumb during the MI period.

To assess to which extent this encoding is effective, BandFocusNet was re-trained and evaluated using only EEG channels from within the ROIs (10 channels). There was no significant difference observed in the model's performance (paired *t*-test; *t*(15) = −0.42, *p* = 0.68).

## IV. Discussion

This study demonstrates the potential to distinguish the MI activation of a supernumerary thumb from the natural thumb at the single-trial EEG level in a VR environment. The performance of BandFocusNet surpassed three benchmarked deep-learning models for EEG data classification, achieving an average accuracy of 70.90% using leave-one-subject-out cross-validation. The model was able to achieve this accuracy while at the same time utilizing the least number of trainable parameters. BandFocusNet's trustworthiness was examined by conducting explainability analysis and validating the neural features tBandFocusNet utilizes towards classification.

BandFocusNet outperforms others by effectively integrating spectral, temporal, and spatial features of EEG data, which explains its enhanced performance. Specifically, the model utilizes a three-branched feature extractor that targets the delta, theta, and alpha bands. This design is particularly relevant, as neural cross-validation analysis revealed significant activation in the right and left frontal regions in the delta and theta bands, which effectively distinguishes between the two thumbs. Additionally, a previous study demonstrated the critical role of the alpha band in differentiating the supernumerary thumb during MI [9]. This study observed significant desynchronization in the ipsilateral sensorimotor area in the alpha band during the MI of the supernumerary thumb, with a more widespread desynchronization across the brain compared to the natural thumb. The inclusion of a squeeze-and-excitation attention mechanism further enhances the model's performance by emphasizing the most relevant features. The usage of layer normalization instead of batch normalization could also have contributed positively to the model's performance, particularly helping in leave-one-subject-out generalization [26].

Explainability analysis revealed the importance of the right and left frontal regions in classifying the MI of the two thumbs apart. Neural cross-validation reinforced this finding as significant differences in the delta/theta power were observed in both ROIs. Particularly, an event related synchronization (ERS), was observed in these bands during the MI of the natural thumb, which was not detected during the MI of the supernumerary thumb (see Fig. 3). This is also found in previous literature, where an ERS between 3–5 Hz in the left inferior frontal gyrus was observed during MI of throwing a tennis ball in a VR environment [27]. It is suggested that the role of the inferior frontal gyrus is a higher order forelimb movement control [28]. In another study, an ERS in the frontal theta band was observed during MI of natural fingers movement compared to motor execution, possibly reflecting a reactive inhibition process or otherwise increased mental effort [29]. Further evidence is also reported that correlates increase in the frontal delta power with attention during mental tasks [30], such as MI. In our study's context and in light of the reported literature, attention does not seem to be a plausible explanation, as both MI tasks require similar levels of attention. However, higher-order forelimb movement control and reactive inhibition processes during MI are typically associated with natural, owned body parts. The absence of ERS during the MI of the supernumerary thumb could therefore indicate a lack of physical sense or embodiment for the supernumerary limb.

These findings regarding the involvement of the frontal region during MI enhance the trustworthiness of BandFocusNet's performance.

The multiband temporal convolution block was further examined and compared to other branching configurations. Table III presents a performance comparison of BandFocusNet alongside alternative branching configurations. Experiments conducted to assess the effect of incorporating higher frequency bands (beta and gamma) caused a slight decrease in performance suggesting that these higher frequencies may introduce irrelevant information for the thumb discrimination task (natural vs supernumerary) through MI. Conversely, removing specific frequency bands, such as alpha, theta, or delta, caused only minor reduction in accuracy. This indicates that while all frequency bands contribute to model performance, none of the individual bands (alpha, theta, or delta) appear to be solely critical. Instead, their combined contribution across multiple bands is likely important for the task.

**TABLE III:** Performance Comparison of the Proposed Model With Different Spectral Band Configurations, Showing the Impact of Adding or Removing Frequency-Specific Branches

**Model**	**Accuracy (%)**
$\delta \theta \alpha$-BandFocusNet (Proposed)	70.9 $\pm$ 8.54
$\delta \theta \alpha \beta$-BandFocusNet	69.7 $\pm$ 8.49
$\delta \theta \alpha \beta \gamma$-BandFocusNet	69.8 $\pm$ 8.13
$\delta \theta$-BandFocusNet	$70.5 \pm 7.93$
$\delta \alpha$-BandFocusNet	$70.4 \pm 7.59$
$\theta \alpha$-BandFocusNet	$69.5 \pm 7.11$

Nomenclatures: $\delta$: Delta Band, $\theta$: Theta Band, $\alpha$: Alpha Band, $\beta$: Beta Band, $\gamma$: Gamma Band.

**Fig. 4.**
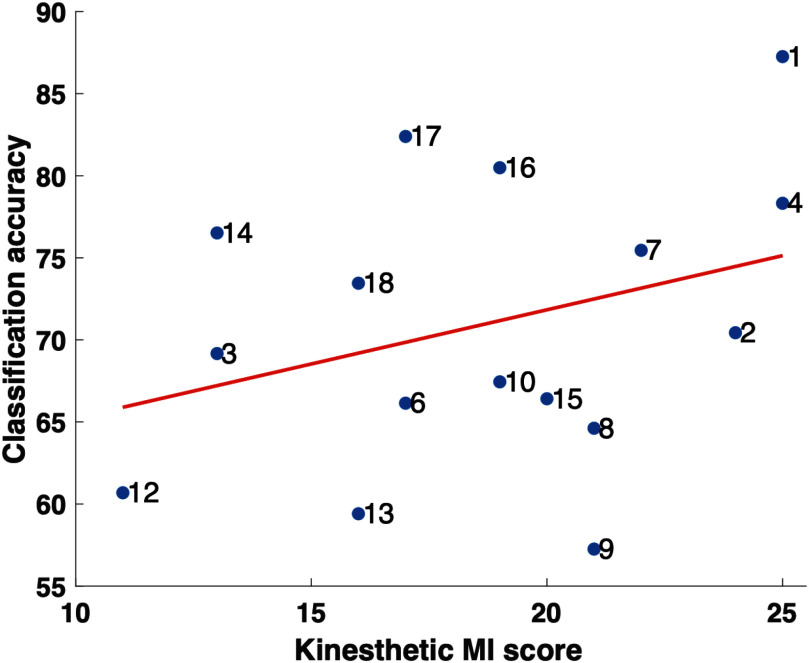
Relationship between the kinesthetic MI score and the classification accuracy based on the KVI questionnaire. Labels of each point represent the subject's number.

Further examination of the model's performance and its relationship with the subjects' kinesthetic MI scores (obtained from the KVI questionnaire [31]) is presented in Fig. 4. The figure shows a correlation plot between the kinesthetic MI score, which represents the MI skill of participants, and classification accuracy. We found no correlation between these two variables (*t* = 1.26, *p* = 0.22, Pearson's *r*(16) = 0.33, CI [−0.20, 0.71]). It is important to acknowledge that other factors may contribute to variations in accuracy. This includes physical attributes such as electrode connectivity, gel placement, and hair condition, as well as personal attributes like the individual's ability to perform MI for a new or foreign body part. Additionally, the KVI questionnaire relies on self-reported data, which may not always be an accurate reflection of the participant's true MI abilities. These influences become particularly evident when examining the variability in classification accuracy between subjects, as shown in Table I. For instance, subjects such as 1 and 17 achieved relatively higher classification accuracy (86.26% and 82.39%), whereas subjects like 9 and 13 exhibited lower accuracy (57.25% and 59.41%).

Despite the classification performance being far from perfect, the current study is well situated to offer new insights compared to the state-of-the-art EEG-based classification of MI for a supernumerary thumb. Previous literature, such as the 4-week longitudinal study by Liu et al. [7], reported a within-subject classification accuracy of 70% in distinguishing MI from rest periods. Another study demonstrated classification of MI signatures for a supernumerary finger, achieving accuracies of 86.3% and 88.2% in distinguishing them from MI of right-hand movement and rest, respectively, using k-fold cross-validation [22]. However, the limitations of these studies lie in comparing the MI signature of the supernumerary effector with either a resting state, which is too simplistic, or a larger limb than the hand, which is not equivalent. In contrast, our study compares the MI of the supernumerary thumb with that of the natural thumb which is a more appropriate comparison. Furthermore, while the referenced studies employed within-subject classification or k-fold cross-validation, we adopt a leave-one-subject-out approach, which better evaluates model generalizability but is often avoided due to its difficulty. These methodological challenges we undertake explain the observed decoding performance.

Despite the promising results, several limitations of this study must be acknowledged. First, the study does not include a classification of MI signatures associated with the flexion and extension of the supernumerary thumb. Although this aspect was investigated, the classification accuracy was relatively low (around 60%), rendering the results less meaningful. A longitudinal study with repeated training sessions may be necessary to obtain more distinguishable MI features for directional control of the supernumerary thumb. Another limitation is the size of the dataset (which is currently limited to 20 subjects only); a larger dataset with more participants is likely to improve classification accuracy and the rigor of the neural cross-validation analysis, thus improving generalizability. Finally, this study was conducted in a VR setting (mainly because it facilitates motor observation from a first-person perspective, which is reported to induce a greater sense of embodiment, provide a controlled and customizable environment for motor imagery training, and ensure a safer setting for training and control of larger supernumerary effectors), in which the obtained results might not be directly applicable to a physical supernumerary thumb in the real environment. The absence of sensory feedback, such as weight and physical attachment to the hand, could have an effect on the transfer of MI activity from the virtual to the physical space. A future direction of interest could involve comparing the neural signatures of motor imagery for the supernumerary effector in real and virtual environments to understand the extent to which these results can be generalized.

## V. Conclusion

This study explored the distinction of MI neural activations for the natural and supernumerary thumbs from single-trial EEG data in a VR environment, while proposing a deep learning model that considers the temporal, spectral and spatial features of EEG data. The proposed model achieves a classification accuracy of 70.9% using the leave-one-subject-out cross-validation method, thus validating the ability of the model at generalizing to novel test subjects. Explainability analysis showed that the proposed model is mostly influenced by the frontal region channels. Further neural cross-validation using ERSPs analysis showed that the there exist a statistically significant differences in the delta and theta bands in the right and left frontal ROIs during the supernumerary and the natural thumbs MI, possibly indicating a lack of embodiment for the supernumerary thumb.

## Supplementary Materials

The supplementary materials include the all the details related to the Materials and Methods section, including the experimental setup and protocol, architecture of BandFocusNet, and the explainability analysis.

Supplementary Materials

## Author contributions statement

 

ME led the study and, along with HA, RV, HS, SA, and FK, conceived and developed the protocol. HA conducted the experiment. HA and JH analyzed the data, with HA and AS performing the machine learning analysis. All authors contributed to discussing the results, writing, and reviewing the manuscript.

## Conflict of Interest statement

The authors declare that there is no conflict of interest regarding the publication of this manuscript.
