# Supplementary material for: BandFocusNet: A Lightweight Model for Motor Imagery Classification of a Supernumerary Thumb in Virtual Reality
Source: IEEE Open J Eng Med Biol. 2025 Feb 3;6:305–11. doi: 10.1109/OJEMB.2025.3537760 (PMC11875636; doi:10.1109/OJEMB.2025.3537760)
Supplement: Supplementary Materials [file supp1-3537760.pdf]

# Supplementary Materials

## BandFocusNet: A Lightweight Model for Motor Imagery Classification of a Supernumerary Thumb in Virtual Reality

Haneen Alsuradi*, Joseph Hong, Alireza Sarmadi, Robert Volcic, Hanan Salam, S. Farokh Atashzar, Farshad Khorrami, Mohamad Eid*

### I. Materials and Methods

#### A. Participants

TWENTY participants, 14 males and 6 females, aged between 18 and 50, took part in this study. All participant were right-handed and performed the task with their right hand. They all had normal or corrected-to-normal vision. Exclusion criteria were being under 18, left-handed, or having a history of traumatic brain injury, neurological disorders, or muscle atrophy. All participants signed a consent form as per the Institutional Review Board (IRB) ethics, with the study being approved by the New York University Abu Dhabi's IRB (HRPP-2023-60). Participants received subsistence allowance for their participation. Four participants were excluded due to poor electrode connections or cap misalignment caused by VR headset movement.

#### B. Experimental setup and protocol

**Pre-Experiment Survey**. Participants first performed a pre-experiment survey to assess their MI skills. This consisted of the kinesthetic visual imagery (KVI) questionnaire which is a widely recognized tool for evaluating the visual and kinesthetic aspects of an individual's MI capabilities [1]. The experimenter described and performed five different body movements, and participants where requested to perform visual MI and kinesthetic MI once for each of the movements. Participants were assisted to self-evaluate the clarity and vividness of their MI through a well-defined scale. For each modality, a score out of 25 was calculated which is distributed across the five movements. We call these scores as the visual MI score and the kinesthetic MI score.

**Main Experiment**. Once done, participants were seated on a comfortable chair and had their arms rested on an arm support placed on a table. They wore the EEG cap and conductive Ag/AgCl gel was put underneath each of the 64 active electrodes. The VR headset (Meta Quest Pro[1]) was placed on top of the EEG cap while minimizing cap displacement. The BrainAmps Standard amplifier[2] (Brain Products GmbH, Germany) was used for data amplification and data were recorded at a 1 kHz sampling rate; see Figure 1a for the setup. Electrodes were positioned following the 10-20 international system. FPz electrode was used as the ground electrode while FCz as the online reference.

[1]https://www.meta.com/quest/quest-pro/

[2]https://brainvision.com/products/brainamp-standard/

The experiment was conducted in VR and consisted of 10 sessions, with the first session dedicated to training participants on the task. At the beginning of each session, participants performed an embodiment exercise where they had to pop virtual balloons with their right hand for 30 seconds. Next, the virtual supernumerary thumb was introduced and participants were instructed to use it to pop balloons with it for another 30 seconds. Figure 1b shows the appearance of the virtual hand equipped with the supernumerary thumb. After the embodiment period, 16 trials were presented and distributed among four conditions: (1) the flexion of the natural thumb, (2) the extension of the natural thumb, (3) the flexion of the supernumerary thumb, and (4) the extension of the supernumerary thumb. Participants were instructed to rest their arms on the table with their palms facing them. A trial started with a baseline period, where the virtual hand was shown to be in a static state for 2 seconds, followed by a motor observation period of another 2 seconds, during which a pre-recorded movement of either the natural or the supernumerary thumb performing flexion or extension was shown. The virtual hand then disappeared and re-appeared in the corresponding pose. This was followed by another baseline period for 2 seconds, during which a fixation cross was shown in front of the palm. The cross then vanished, indicating the start of the MI period, where participants were asked to engage in MI of the previously observed movement; during this time the virtual hand and supernumerary thumb were static. Following the MI period, participants were asked to rate their success in performing the MI task using a keypad with their left hand (binary rating). Thus, each participant completed 160 trials, with the initial 16 trials conducted during the training session. Figure 1c shows the timeline of the experimental task.

**Post-Experiment Survey**. After the main experiments, participants were asked to fill a post-experiment survey to capture aspects of their experience, which detailed their self-reported MI performance for both the natural and supernumerary thumbs, as well as the embodiment of the supernumerary thumbs in the VR environment.

#### C. EEG data processing

EEG data were pre-processed offline using MATLAB release 2022a (MathWorks, United States) and EEGLAB toolbox [2] (v14.1.2). EEG data were minimally preprocessed to allow the deep learning models to capture relevant information automatically. The first step was bad electrodes removal; four electrodes (FT9, FT10, TP9, TP10) were discarded because they are

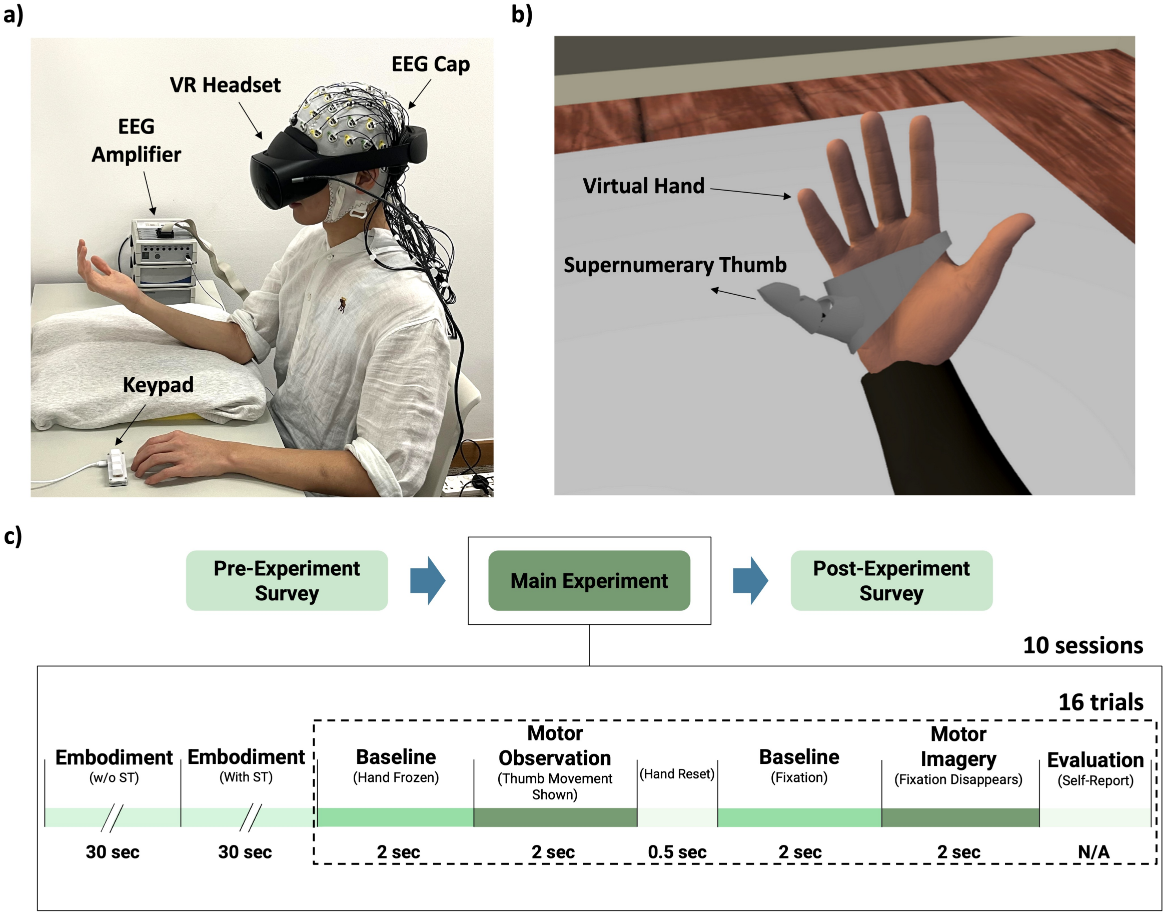


Fig. 1. Overview on the setup and protocol. (a) The main experiment experimental setup including the EEG acquisition system and the VR headset (b) Snapshot of the virtual hand participants could see with an attached supernumerary thumb. (c) An illustration of the experimental protocol with the timeline of the experimental tasks.

located outside the brain's area, near the ears, and another four (T7, T8, FT7, FT8) were excluded due to contamination from electromagnetic interference caused by the VR headset battery. Next, EEG data were band pass filtered between 0.1–80 Hz and a notch filter was applied at 50 Hz to suppress power line noise. A lenient data cleaning methodology was applied using the Artifact Space Reconstruction (ASR) method [3], to mitigate ocular and muscular artifacts, while retaining most of the neural activation. This was done by setting the standard deviation cutoff for removal of bursts (argburst) to 20, which has been shown to effectively clean EEG data from ocular artifacts while retaining the neural features of the data [4]. This was followed by common average referencing and retaining the online reference channel data, FCz. Lastly, EEG data were epoched to 2 seconds of the MI period, based on the duration of interest. Data used for training and validating the deep learning models were downsampled to 200 Hz.

For the neural-cross validation, further processing steps were taken. EEG data were transformed to the time-frequency domain calculating the event related spectral perturbations (ERSPs). This was done using Morlet wavelets transform, where the wavelets are defined as complex sine waves tapered by a Gaussian. The wavelet's frequency ranged from 1–80 Hz, with linear spacing. The power of the transformed epochs was then calculated by squaring their amplitude. Next, the trials of the same condition were averaged per subject such that flexion and extension of the same thumb were considered as one condition. The ERSPs went through decibel conversion and their power were normalized with respect to the baseline following the formula: $10\log_{10}\left(\frac{\text{activity}}{\text{baseline}}\right)$. Baseline normalization was applied to the data using a baseline window from -1.25 to -0.25 seconds relative to the onset of the MI period.

### *D. BandFocusNet model*

EEG signals are known for their complexity, with brain activity reflected across multiple frequency bands and spatial locations. BandFocusNet was thus designed to effectively capture the temporal, spectral, and spatial characteristics of EEG data. Particularly, the model design is grounded in neuroscience literature of MI, where it is reported that activation in the delta, theta, and alpha bands are present [5]–[7].

The architecture, illustrated in Figure 2, comprises three main components: (1) a Temporal Convolution block, (2) a Channel Attention (Squeeze and Excitation) block, and (3) a Spatial Convolution block.

**Temporal Convolution Block**. The temporal convolution block leverages the multi-band nature of EEG signals by

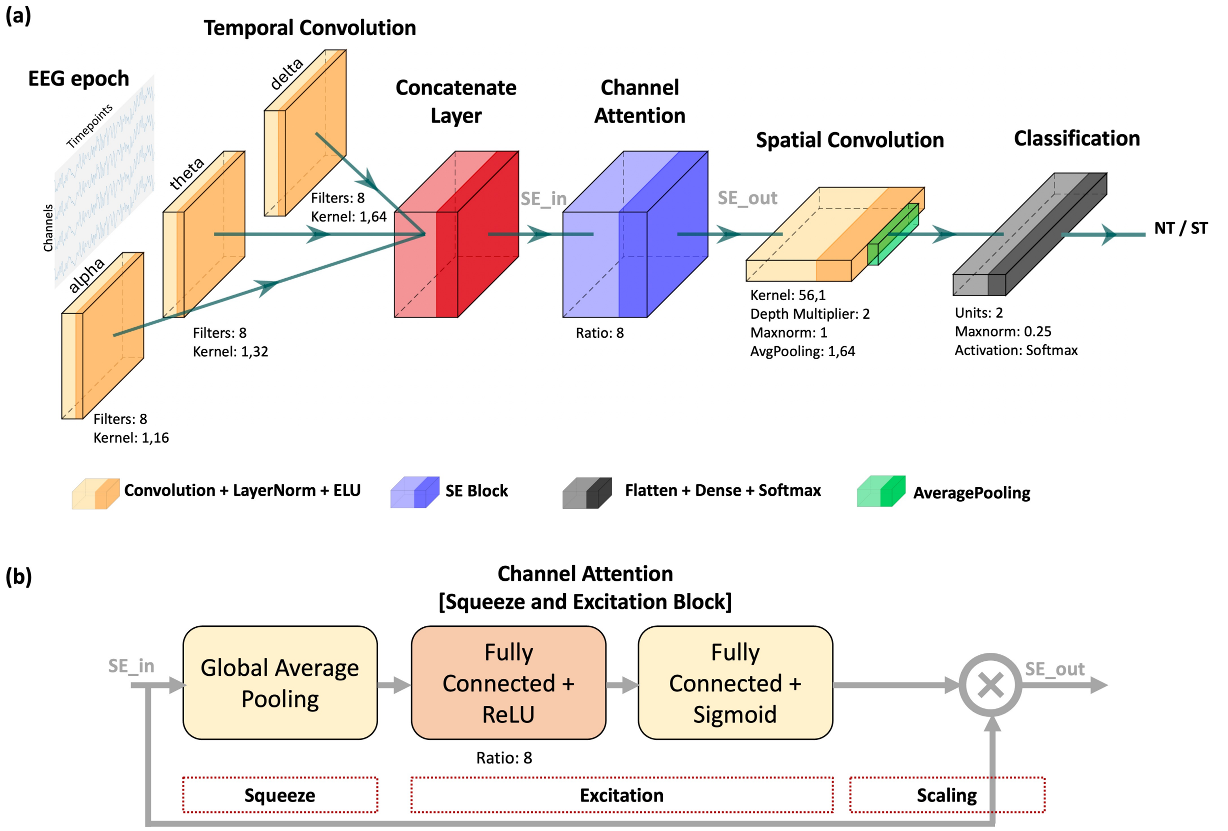


Fig. 2. BandFocusNet model. (a) The architecture of the proposed model, with a breakdown of the role of each block. (b) An illustration of the channel attention block (squeeze-and-excitation).

employing three distinct convolutional branches, each designed to capture temporal features from different frequency bands: delta, theta, and alpha. These bands were specifically chosen because they represent key frequency ranges commonly associated with different cognitive and physiological states which are relevant to MI [5]–[7]. Each branch processes the same input epoch but with distinct kernel size, thus targeting specific frequency band. The first branch, with a large kernel size of 64, extracts features from the delta band (0.5–4 Hz), which represents low-frequency brain activity. The second branch focuses on the theta band (4–8 Hz) using a kernel size of 32, while the third branch targets the alpha band (8–13 Hz) with the smallest kernel size of 16. Note that the kernel size is calculated using the formula: kernel size $= \frac{\text{sampling frequency}}{\text{frequency of interest}}$. This multi-scale approach enables the model to capture relevant temporal dynamics across different frequency bands. After convolution, feature maps from the three branches are concatenated along the channel dimension, ensuring that the temporal information from various frequency bands is effectively integrated.

**Squeeze and Excitation Block.** A squeeze-and-excitation technique is employed as an attention mechanism to amplify and diminish importance for the more important and less important channels, respectively [8]. This attention mechanism is able to give more weights to the spectral band that is more important towards the classification task. The squeeze-and-excitation block consists of three processes (see Figure 2b): squeeze, excitation, and scaling. In the squeeze process, the feature maps are reduced through global average pooling, such that the feature map is of a dimension equal to the number of channels, $C$. This is equivalent to summarizing each of the channels in the input feature map (with dimensions $H$ and $W$ for height and width, respectively) into a single unit, $z_c$:

$$z_c = \frac{1}{H \times W} \sum_{i=1}^{H} \sum_{j=1}^{W} x_{i,j,c} \tag{1}$$

where $x_{i,j,c}$ is the value of the feature at position $(i,j)$ in the $c_{th}$ channel.

This is followed by a dense layer that translates the feature map of size $C$ to a feature map of size $C/ratio$. Another dense layer is added where the feature map dimension is again converted to size $C$. These two dense layers are used to learn and extract channel weights, $s$:

$$s = \sigma(W_2 \delta(W_1 \mathbf{z})) \tag{2}$$

where $W_1$ and $W_2$ are the weights of the dense layers, $z$ is the squeezed channel features, $\delta$ is the ReLU activation function and $\sigma$ is the sigmoid activation function. Once channel weights are extracted, the input to the squeeze-and-excitation block is multiplied by these weights, thus, adjusting the importance of each channel:

$$\hat{x}_{i,j,c} = s_c \cdot x_{i,j,c} \tag{3}$$

where $\hat{x}_{i,j,c}$ is the calibrated feature map value at position $(i,j)$

for channel $c$.

**Spatial Convolution Block.** Next is a depth-wise convolution layer that is responsible for capturing spatial features, given the kernel shape is vertical, thus extracting spatial interdependencies between channels. In depth-wise convolution, each channel is convolved with a different kernel [9]. This design choice was intentional as different channels carry information from different frequency bands. The use of depth-wise convolution permits flexibility in learning features differently for the different bands. An average pooling layer is added with a pooling size of 64, further reducing the dimensionality in the time domain. Lastly, the classification block is comprised of a dense layer of two units, with a max-norm regularization of 0.25 imposed on the layer weights. A softmax activation function is used to normalize the output probabilities of the two classes: the natural and supernumerary thumbs.

It can be observed from Figure 2a that a LayerNorm layer is added after every convolutional layer. This is slightly different from the usual practice of adding BatchNorm layers. The rationale behind this choice is the several advantages that the LayerNorm offer for the problem under study. Layer normalization is more appropriate for tasks such as sequence modeling (as seen in EEG data, which are time-series), for training with relatively small batch sizes (like our use of a batch size of 16), and in scenarios where covariate shifts occur (for instance, when the distribution of training and testing data differs, which is common in leave-one-subject-out testing where the test data comes from a completely new subject) [10].

### E. Baseline models

To demonstrate the effectiveness of BandFocusNet, we tested the dataset with three state-of-the-art models for the purpose of comparison. Those models are the EEGConformer [11], CapsuleNet [12] and ATCNet [13]. The EEG Conformer is a compact convolutional transformer model designed for EEG classification, which consists of a convolution module to capture low-level local features and a self-attention module to extract global correlations. CapsuleNet on the other hand employs capsules as primary building blocks in the deep model, resolving some of the CNN's drawbacks such as the inability to accurately extract hierarchical relationships [12]. ATCNet is a deep learning model that utilizes attention-based temporal convolutional networks, developed specifically for EEG-based MI classification. All of these models showed efficacy in the literature in EEG data decoding. The performance and the number of trainable parameters of these models are compared to our BandFocusNet.

### F. Training and validation

We employed the leave-one-subject-out cross-validation method due to its ability in assessing the models' generalization performance across different subjects [14]. All models were trained for 30 epochs with a batch size of 16 and a learning rate of $5 \times 10^{-5}$ and a weight decay of 0.01. We also employed Adam optimizer to minimize the cross-entropy loss function, which is commonly used to optimize classification models by measuring the difference between predicted probabilities and actual class labels. All experiments were conducted using Keras 2.10 with an NVIDIA A100 GPU.

### G. Explainability analysis

Explainability analysis is used to assess the features that the model relies on towards its prediction. In this study, we used Shapley values to examine the most influential features in predicting the thumb (natural vs supernumerary) for which the MI task is performed, using BandFocusNet. This analysis confirms whether the model is picking up on features that are explainable from a neural perspective, or otherwise relying on superficial features with no neural grounds. For every input feature, a Shapley value is calculated that represents the importance of that feature. For the calculation of the Shapley value for a given feature, feature sets which comprise all possible coalitions of the $N$ existing features while excluding the feature of interest ($i$) are generated. The worth function, $v$, which in this context is simply the function of the model that is used for predicting an input instance to a particular class, is used to evaluate the worth of feature $i$ by considering coalitions with and without feature $i$. The difference in prediction (between the coalitions with and without the feature of interest) is calculated and represents the marginal contribution of the feature $i$ in the prediction process.

The Shapley value, $\varphi_{i(v)}$, for a feature $i$ evaluated using model $v$ is computed by averaging its marginal contribution over all possible permutations of feature coalitions, as shown in the following equation [15]:

$$\varphi_{i(v)} = \sum_{S \subseteq N/\{i\}} \frac{|S|!(|N|-|S|-1)!}{|N|!}(v(S \cup \{i\}) - v(S)) \quad (4)$$

where $N$ is the set of all features, $S$ is a subset of the features without feature $i$, $|N|$ is the number of all features, $|S|$ is the number of features in the subset $S$, and $v$ is the worth function.

In this study, the input data had two types of features, spatial (consisting of the EEG channels) and temporal (consisting of the time-points for each channel). We used the SHAP package [16] to calculate the Shapley values for all features and all subjects across the two conditions. The absolute Shapley values were then averaged over subjects and conditions, then normalized. We report the absolute scaled Shapley values for BandFocusNet.

Once the Shapley values were extracted, features that were given the largest weights were explored. In particular, we extracted electrodes that were most influential in prediction to define regions of interest (ROI). ERSPs in the defined ROIs are examined to explore if there exists any valid neural activations that encodes the respective thumb.

### H. Statistical analysis

To compare the performance of BandFocusNet using all channels with that of using only channels within the ROI, we employed the Shapiro-Wilk test, which is particularly suitable for small sample sizes [17], to confirm the normality of the accuracy score distributions. After confirming normality, a paired $t$-test was conducted to assess the statistical significance of the performance differences.

## References

[1] F. Malouin, C. L. Richards, P. L. Jackson, M. F. Lafleur, A. Durand, and J. Doyon, "The kinesthetic and visual imagery questionnaire (kviq) for assessing motor imagery in persons with physical disabilities: a reliability and construct validity study," *Journal of neurologic physical therapy*, vol. 31, no. 1, pp. 20–29, 2007.

[2] A. Delorme and S. Makeig, "Eeglab: an open source toolbox for analysis of single-trial eeg dynamics including independent component analysis," *Journal of neuroscience methods*, vol. 134, no. 1, pp. 9–21, 2004.

[3] C. A. E. Kothe and T.-P. Jung, "Artifact removal techniques with signal reconstruction," Apr. 28 2016, uS Patent App. 14/895,440.

[4] C.-Y. Chang, S.-H. Hsu, L. Pion-Tonachini, and T.-P. Jung, "Evaluation of artifact subspace reconstruction for automatic eeg artifact removal," in *2018 40th Annual International Conference of the IEEE Engineering in Medicine and Biology Society (EMBC)*. IEEE, 2018, pp. 1242–1245.

[5] A.-M. Cebolla, E. Palmero-Soler, A. Leroy, and G. Cheron, "Eeg spectral generators involved in motor imagery: a swloreta study," *Frontiers in psychology*, vol. 8, p. 2133, 2017.

[6] F. Binkofski, K. Amunts, K. M. Stephan, S. Posse, T. Schormann, H.-J. Freund, K. Zilles, and R. J. Seitz, "Broca's region subserves imagery of motion: a combined cytoarchitectonic and fmri study," *Human brain mapping*, vol. 11, no. 4, pp. 273–285, 2000.

[7] H. Alsuradi, J. Hong, A. Sarmadi, R. Volcic, H. Salam, S. F. Atashzar, F. Khorrami, and M. Eid, "Neural signatures of motor imagery for a supernumerary thumb in vr: an eeg analysis," *Scientific Reports*, vol. 14, no. 1, 2024.

[8] J. Hu, L. Shen, and G. Sun, "Squeeze-and-excitation networks," in *Proceedings of the IEEE conference on computer vision and pattern recognition*, 2018, pp. 7132–7141.

[9] F. Chollet, "Xception: Deep learning with depthwise separable convolutions," in *Proceedings of the IEEE conference on computer vision and pattern recognition*, 2017, pp. 1251–1258.

[10] J. L. Ba, J. R. Kiros, and G. E. Hinton, "Layer normalization," *arXiv preprint arXiv:1607.06450*, 2016.

[11] Y. Song, Q. Zheng, B. Liu, and X. Gao, "Eeg conformer: Convolutional transformer for eeg decoding and visualization," *IEEE Transactions on Neural Systems and Rehabilitation Engineering*, vol. 31, pp. 710–719, 2022.

[12] S. Sabour, N. Frosst, and G. E. Hinton, "Dynamic routing between capsules," *Advances in neural information processing systems*, vol. 30, 2017.

[13] H. Altaheri, G. Muhammad, and M. Alsulaiman, "Physics-informed attention temporal convolutional network for eeg-based motor imagery classification," *IEEE Transactions on Industrial Informatics*, vol. 19, no. 2, pp. 2249–2258, 2022.

[14] M. P. Pauli, C. Pohl, and M. Golz, "Balanced leave-one-subject-out cross-validation for microsleep classification," *Current Directions in Biomedical Engineering*, vol. 7, no. 2, pp. 147–150, 2021.

[15] S. Lipovetsky and M. Conklin, "Analysis of regression in game theory approach," *Applied Stochastic Models in Business and Industry*, vol. 17, no. 4, pp. 319–330, 2001.

[16] S. M. Lundberg and S.-I. Lee, "A unified approach to interpreting model predictions," in *Advances in neural information processing systems*, 2017, pp. 4765–4774.

[17] B. Yazici and S. Yolacan, "A comparison of various tests of normality," *Journal of statistical computation and simulation*, vol. 77, no. 2, pp. 175–183, 2007.
